# Experimental Study on the Correlation between Crack Width and Crack Depth of RC Beams

**DOI:** 10.3390/ma14205950

**Published:** 2021-10-10

**Authors:** Yue Li, Juhui Zhang, Zhongguo Guan, Youliang Chen

**Affiliations:** 1 Department of Civil Engineering, University of Shanghai for Science and Technology, Shanghai 200093, China; 181520110@st.usst.edu.cn (Y.L.); ylchen@usst.edu.cn (Y.C.); 2 Department of Bridge Engineering, Tongji University, Shanghai 200092, China; guazhongguo@tongji.edu.cn

**Keywords:** load, beam, crack width, crack depth, correlation

## Abstract

The depth of cracks propagating inside reinforcement concrete (RC) components is barely able to be detected by visual inspection. Without any help from facilities, crack width can provide us with a proper way to explore the depth of cracks developing. Therefore, this paper tried to explore the correlation between crack width on the surface and crack depth. A static loading test was conducted on eight RC beams, considering the variation of concrete strength, cover, and reinforcement ratio. The test results indicate that concrete strength has a certain impact on cracking load and the propagation of cracks is mainly related to reinforcement ratio. The linear changes in load and crack width can be found. Originally, crack depth markedly increased with loading, but when restricted by compression zone of concrete and the height of beams, crack depth stopped extending finally. The correlation between crack width and crack depth was analyzed by studying work phases of a cross-section and experimental data. The fitting function achieved in this paper was determined to be a good agreement between the theoretical and the experimental relationship.

## 1. Introduction

The serviceability of concrete structures is a primary concern among current design specifications, especially for members in coastal zones and severe or aggressive environments. For mass reinforced concrete (RC) constructions, cracks cannot be avoided, and thus constructions are not crack-free in real situations. This will pose a great threat on the service life and reliability of buildings. Cyclic loading and salt water could cause a great loss of capacity and the most obvious sign of this deterioration is the emergence of cracks [1,2,3,4,5]. Corrosive ions from external environments can penetrate into RC structures and corrode reinforcements through cracks. With the development of corrosion process, rust would cause concrete tensile stress that may be sufficiently large to induce internal micro-cracking and eventually spalling of concrete cover [6]. In a brief word, cracks can accelerate the process of corrosion and corrosion can promote the development of cracks. Therefore, studying on cracking behavior can be conducive to accurately predict and improve the service life of the project.

Accomplishing adequate research on crack characters are primary before optimizing life-cycle performance of concrete structures or even predicting corrosion initiation. Rasmussen et al. [7] proposed an approach for outlining crack development in beams involving description of the system of cracks and estimation of reinforcement stresses. Rasmussen defined the width of crack as the sum of slip from both sides adjacent to the considered crack, and deduced an analytical approach for the prediction of crack width from summation of steel strains. To accurately describe the geometrical characteristics of shrinkage cracks, Zhu et al. [8] observed the whole process of shrinkage cracking in concrete and analyzed cracks statistically. The experimental results showed the depth-to-width ratio and cracks tip angle were approximately 44.3° and 2.6°, respectively. Laterza et al. [9] proposed an analytical model for describing the stress state provoked by a confining rectangular hoop, also taking into account the effects of additional external strengthenings. The model is capable of describing the confinement state within the section core at any increment of axial strain, and the constitutive principle of cracking is well explored. Naotunna et al. [10] applied an axial tensile load on specimens, and the generated cracks were sealed by using epoxy. Then, the cracked specimens were cut and the crack width propagation along the concrete cover depth observed. They found that specimens with ribbed bars behave in a way more related to the no-slip theory, while specimens with smooth bars behave in a manner more related to the bond-slip theory. Tung et al. [11] carried out an analysis of crack development and shear transfer mechanisms in beams with a low amount of shear reinforcement. The investigation was analyzed using a group of appropriate equilibrium conditions, geometric conditions, and constitutive relationships. A simplified model for the shear resistance of rectangular beams was obtained and the validated analysis can be used to investigate the relative contributions of different shear resisting mechanisms during the shear crack development. Cheng et al. [12] presented a 2D diffusion-mechanical model for concrete cover cracking caused by non-uniform corrosion of reinforcement. The model showed that rebar diameter has limited influence on the cracking patterns, but smaller diameters will result in longer crack initiation, extended through time, and an increase in surface crack width. Yang et al. [13] developed a numerical method to predict concrete crack width for corrosion-affected concrete structures. A cohesive crack model for concrete was implemented in the numerical formulation to simulate crack initiation and propagation and the surface crack width was obtained as a function of service time.

The standards of each nation have different control ranges and requirements in crevice of concrete building. The bond-slip and no-slip theory are employed to calculate crack width by the British Standard Institution (BS) [14] and Eurocode (EN) [15]. The maximum crack width is specified on the basis of the different working conditions and purposes of structures. For example, for buildings with aesthetic needs, the maximum crack width is 0.1 mm; for buildings in good conditions which are used indoors, the maximum crack width can be 0.4 mm. Due to the discrete characteristics of concrete, the actual crack widths cannot be accurately calculated. Therefore, the American Concrete Institute (ACI) [16] no longer adopts this method for directly calculating the crack width of components. Instead, their method limits the spacing of reinforcement bars to control the crack widths. In essence, the expression of maximum crack width is established according to the no-slip theory. On the other hand, referring to the specifications of ACI, Frosch [17] proposed the expression of maximum crack width (shown in Table 1). Under sufficient statistics and comprehensive theory of bond-slip and no-slip, the Chinese code (GB) [18] decides that the first step is to determine the average crack spacing and crack width under short-term loads. Then, they consider the effects of long-term load. The model is a half-theoretical and half-empirical formula. Table 1 summarizes the calculation formulas for crack width in each norm and Table 2 compares the maximum allowable crack width of buildings in Europe and China.

The crack width on the surface of components is easy to measure, but the internal cracking is hard to detective without using any apparatus. Major attention in the literatures and construction standards is paid to crack width, while the importance of crack depth is often ignored (let alone the relation between width and depth). In order to provide a theory for assessing the reliability of structures in fatigue or corrosive environments, the objective of this paper is to analyze the correlation between crack width and crack depth in beams. To realize this purpose, eight beams with different concrete strength, concrete cover thickness, and reinforcement ratios were simply supported in three-point-bending tests. The crack width and crack depth during loading were registered and discussed in this paper. All specimens and loading systems were designed in accordance with design codes and guidelines for RC elements [18,19].

## 2. Experimental Program

### 2.1. Materials and Preparation of Specimens

Ordinary Portland cement 42.5 (Youhang Building Materials Co., Ltd., Shanghai, Shanghai, China) was in usage and the coarse aggregate (Youhang Building Materials Co., Ltd., Shanghai, China) size was from 5 to 25 mm. The concrete mix proportions are presented in Table 3. The fresh concrete was poured into the 9 cubic and 9 prism wooden molds and then placed in a standard curing chamber for 28 days. The longitudinal reinforcement with different diameters was applied in tests. The yield strength (*f_y_*) and ultimate strength (*f_su_*) of reinforcing steels (Weibo Industry and Trade Co., Ltd., Xuzhou, China) were tested in accordance with the Standard [19] and the modulus of elasticity (*E_s_*) in this paper is 2 × 10^5^ N/mm^2^. The average compressive (*f_cu_*) and splitting tensile strength (*f_tk_*) of the concrete after curing are shown in Table 4, including mechanical characteristics of reinforcements in beams. Table 5 shows the details of properties of the beams. Three different concrete strength and protective layer thickness were taken into consideration. The type of reinforcement in tensile zone was HRB400 and the diameter ranged from 14 to 22 mm.

**Figure 1:**
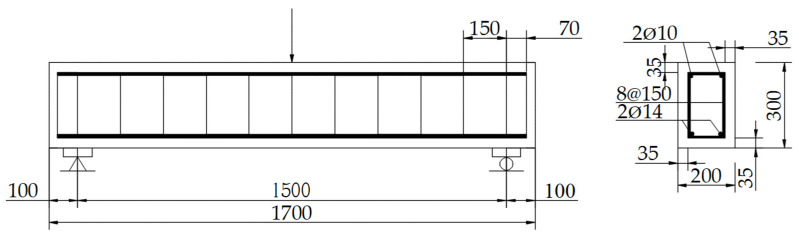
Geometric details of beam A (mm).

Beam A was taken as a reference and its reinforcement layout is shown in Figure 1. The dimension for all the RC beams was 200 × 300 × 1700 mm^3^, and effective span was 1500 mm. Stirrups with spacing of 150 mm were embedded to avoid shear failure.

### 2.2. Loading System

**Figure 2:**
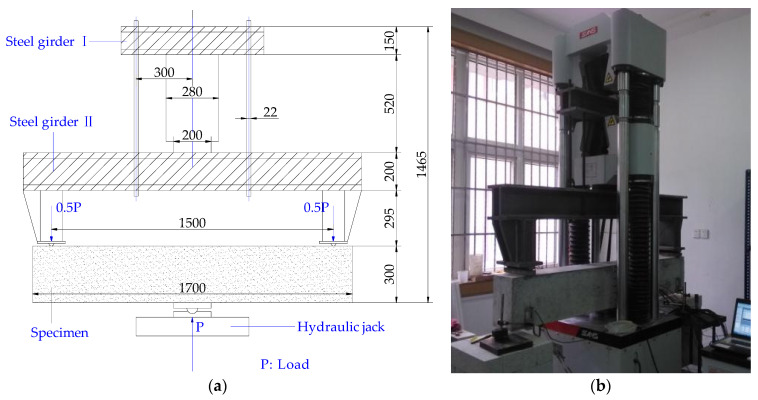
Test loading device: (**a**) schematic diagram of the test setup; (**b**) overview of the test setup (mm).

Beams were simply supported and subjected to reverse three-point-bending (SHT4106G Microcomputer-controlled Electro-Servo Universal Tester, Mechanical Testing & Simulation, Eden Prairie, MN, USA). This is to ensure the emergence of cracks was on the top surface of beams, for observation conveniently (see Figure 2). By lifting the platform, the hydraulic jack applied loads on beams with force control at a rate of 1 kN/min. For making necessary records, like taking photos (MG10085-1A Reading Microscope, Haishu Dayu Test Instruments Co., Ltd., Ningbo, China) and noting the depth, the loading machine was kept statically for 5 min after every 2 min’s loading.

### 2.3. Measurement

**Figure 3:**
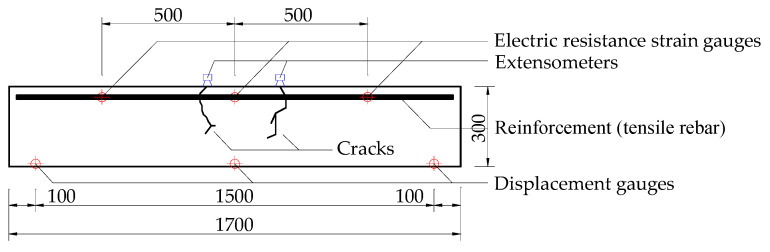
Layout of strain and displacement gauges (mm).

Electric resistance strain gauges (Rhythm Technology Co., Ltd., Shanghai, Shanghai, China) were used to measure the strains in the longitudinal reinforcements. Six gauges in total were placed on two tensile rebars (one at the mid-span and another two at each side of mid-span with a distance of 500 mm, as shown in Figure 3). Displacement gauges were installed underneath the beams to monitor the vertical deformations and settlements during tests. The output was logged into a high-speed data acquisition system: DH3816N-2015 (Donghua Technology Co., Ltd., Taizhou, China).

The locations of crack initiating on the surface are indeterminate due to concrete heterogeneous system [20]. The first crack and the second one must be found pretty carefully and patiently. Once the cracks were checked out, they would be kept under constant surveillance by extensometers. Crack width was observed and read by a microscope. Experimenters had been keeping eyes on the development of crack and recording related data during loading.

## 3. Results and Analysis

### 3.1. Cracking Behaviors

The first and second cracks of beams began to appear on the top surface one after another as the load increased. The initial crack width was normally 0.02 mm, which is the minimum value can be found out by the naked eye. At the moment of the first crack emerging, no visible crack appeared on side-surface of RC beams. Table 6 shows the cracking load to each specimen. It can be roughly determined that Beam G with the highest concrete strength had the maximum cracking load, indicating that the splitting tensile strength of concrete has a certain influence on the cracking load.

**Figure 4:**
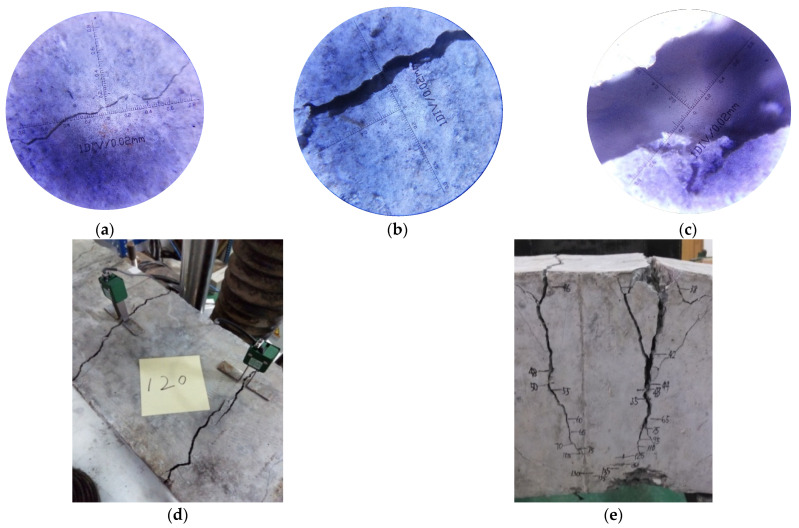
Cracking pattern of beam A1: (**a**) crack initiation; (**b**) crack propagation; (**c**) crack final state; (**d**) crack width on top surface; (**e**) crack depth on side surface.

For RC beams, the initial length of first two cracks was less than 80 mm and the mean crack spacing was 155 mm. Figure 4 shows the development process of cracks of A1. In all the cases, more and more transverse cracks showed up as the applied load increased, and they were perpendicular to edges of the beams. During the processes of cracking, even the fracture sounds could be heard. Since specimens were subjected to flexural and shear stresses, scattered cracks on top were assembling at the bottom of the beams. The magnitude of cracks further increased in both width and depth with loading, followed by the crushing of concrete in compression zone. Finally, the load suddenly dropped after beams reaching the ultimate capacity.

### 3.2. Load and Crack Width

**Figure 5:**
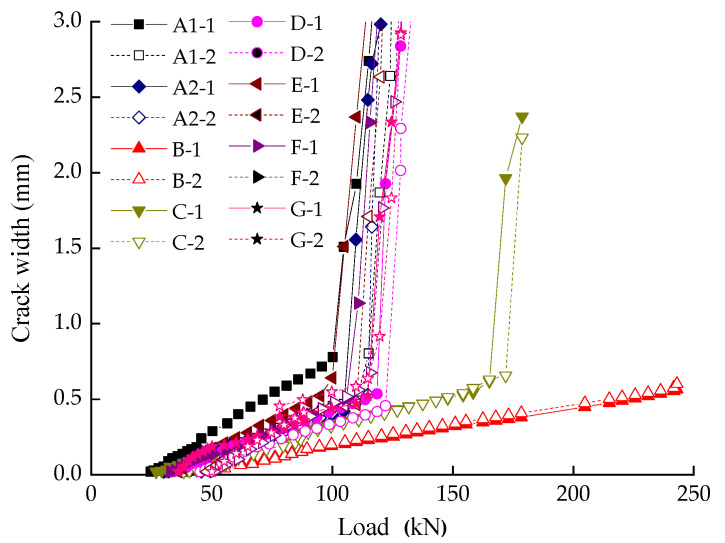
The relation between load and crack width. A1-1: The first crack of beam A1, et cetera. G-2: The second crack of beam G.

The opening width of the first two cracks was monitored with clip extensometer produced by American Epsilon Company (Jackson, MS, USA). The measured data of crack width and applied load are plotted in Figure 5. Note that, for safety, the load of Beam B was withdrawn when it was beyond 250 kN, because Beam B was over-reinforced.

**Figure 6:**
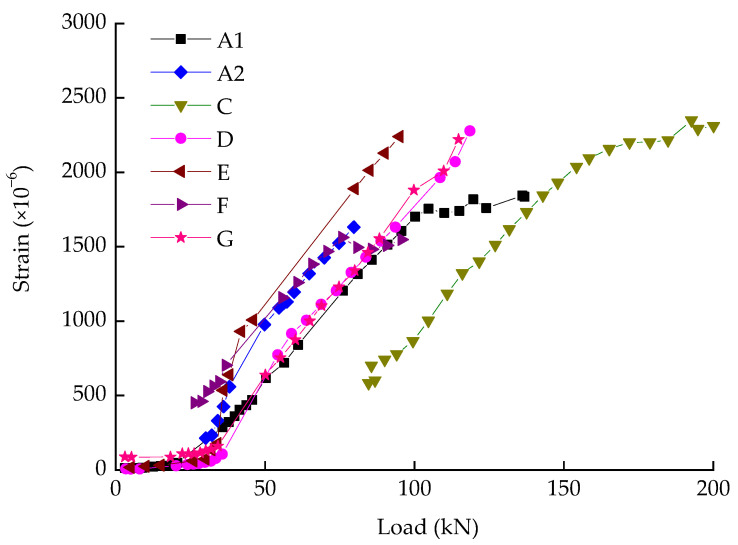
Load-strain curves of beams.

The width-load curves get a similar developing trend in Figure 5. As the load increased to 30 ± 4 kN and 46 ± 2 kN, successively, the first two cracks of each beam showed up on the top surface. In this stage, the deformation of beams was mainly caused by elastic deformation of aggregates and cement crystals. When the applied load reached 75–85% of ultimate loading capacity, the turning points arose, where the crack width ranged from 0.5 to 0.8 mm (see in Figure 5). The turning point (a sign of yielding) can be viewed as the starting point of crack unstable propagation, which is depending on yielding strength of longitudinal reinforcements (shown in Figure 6). The results show a linear increase in crack width with the load during the entire procedure. The comparison between beams illustrated that the parameter of reinforcement ratio had a major impact on crack width, which has been widely discussed in the literatures [21,22,23,24]. These studies commonly agree that crack width decreases with the increase of the reinforcement ratio, which is consistent with the experimental results obtained in this paper.

In order to verify the reliability of the test, we compared the reinforcements stress obtained by two approaches. One method is to calculate the stress through crack width, and the other is to calculate the reinforcements stress via the measured data from electric resistance strain gauges (the slope of curves in Figure 5).

Under long-term load, non-uniform shrinkage strains [25], creep behavior [26] and bond-slip [27] can increase average strain of tensile reinforcement and then widen cracks existing in RC members. The maximum crack width, caused by long-term load, can be obtained using Equation (1) according to the Code [18].

(1) $$w_{\max}=\tau_{l}w_{s,\max},$$

where *w*_max_ is the maximum crack width caused by long-term load, *w_s_*_,max_ is the maximum crack width induced by short-term load, and *τ_l_* is the crack expansion coefficient.

Note that the load applied in this paper was regarded as short-term load. For a RC beam with rectangular cross section, the *w*_max_ of that beam can be calculated form GB (see Table 1) under the quasi-permanent combination of actions by considering the long-term action effect.

*ψ* and *ρ_te_* can be counted via Equations (2) and (3), respectively.

(2) $$\psi =1.1-0.65\frac{f_{tk}}{\rho_{te}\sigma_{sq}};$$

(3) $$\rho_{te}=\frac{A_{s}}{A_{te}},$$

where *f_tk_* is the tensile strength of concrete (represented in Table 4), and *A_s_* and *A_te_* are effective area of steels and concrete in tension, respectively. Here, *A_te_* equals 0.5*bh*.

The figures of parameters mentioned in Equations (1)–(3) and Table 1 are shown in Table 7. Therefore, *σ_sq_* of each beam can be acquired through crack width. 

The stress of reinforcements in tests also can be figured out by Equation (4).

(4) $$\sigma_{a}=E_{s}\varepsilon ,$$

where *σ_a_* is the tensile stress in longitudinal reinforcement, calculated via measured data and *ε* is strain, measured by electric resistance strain gauges. 

The figures of *σ_sq_* and *σ_a_* are list in Table 8. The reinforcement stresses obtained by the two approaches seem to be in a good agreement. 

### 3.3. Load and Crack Depth

**Figure 7:**
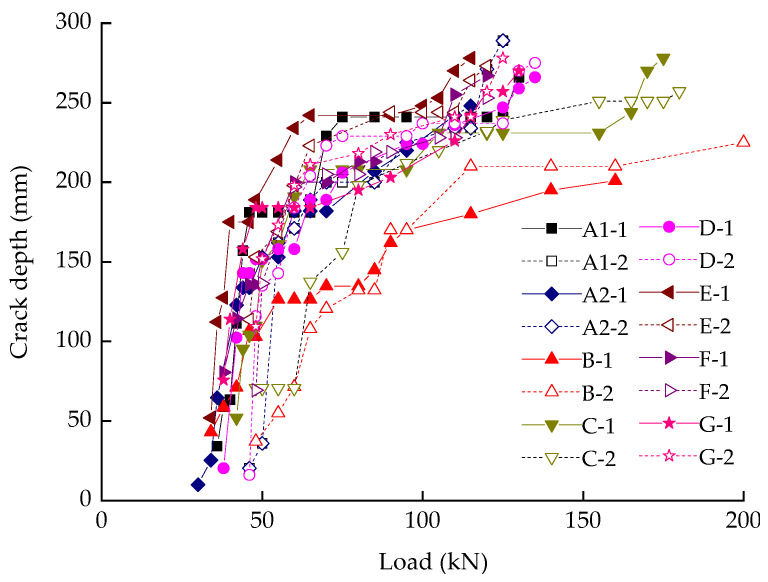
The relation between load and crack depth.

Figure 7 shows the load-crack depth curves of all the tested beams. Originally, crack depth developed at a fast rate and the load-crack depth curves are almost vertical to the horizontal axis. Once the crack depth exceeded the original position of the neutral axis (i.e., half the height of the test piece), the crack extending rate was getting reduced because the compression zone limited its growth. With cracking decreasing the effective area of concrete in tensile zone, the neutral axis was moving towards compression zone. Therefore, the crack extending rate was changing in accordance with the moving rate of neutral axis, which became slower in the later. 

The results in Figure 7 indicate that the development of crack depth is related to the reinforcement ratio and concrete cover thickness. From the curves of Beam B and C, a higher reinforcement ratio and thicker concrete cover mean lower crack depth extending rate. The cover thickness was regarded as a need to transmit tensile stresses generated at the bar-concrete interface to the effective concrete area surrounding the bar [28]. Moreover, the cover of concrete is not only related to the internal cracks, but also influence the time to the failure of specimens. It could be clearly illustrated from the curves of Beam E (cover: 45 mm) and Beam D (cover: 25 mm) in Figure 7.

## 4. Discussion

### 4.1. Work Phases of Concrete Across-Section and Correlation between Crack Width and Depth

**Figure 8:**
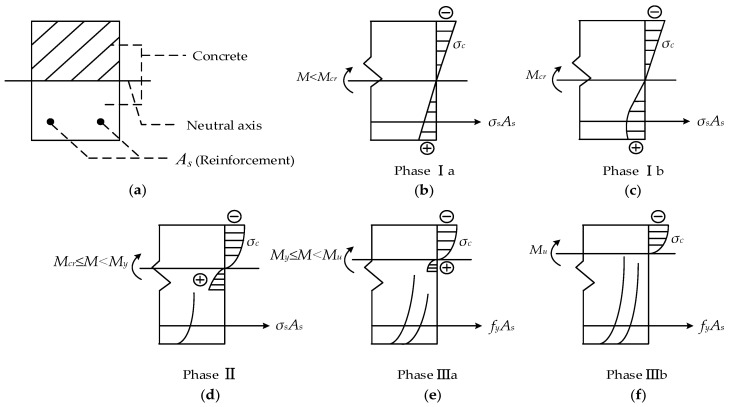
(**a**) A cross-section in the mid-span of a RC beam and its work phases: (**b**–**f**).

**Figure 9:**
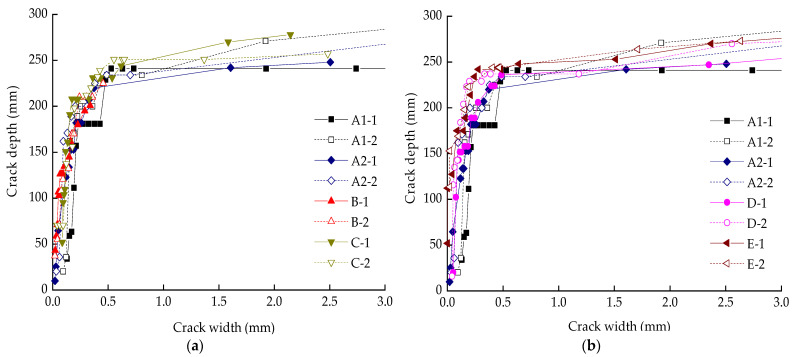

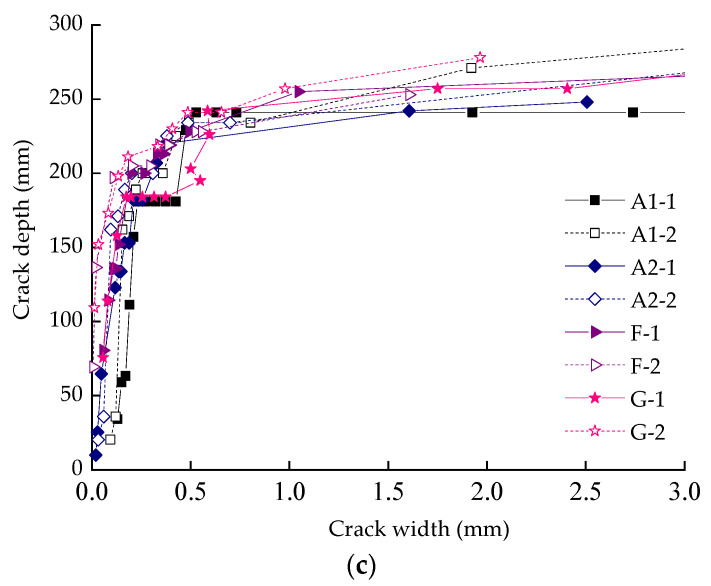
The correlation between crack width and depth with vary (**a**) reinforcement ratio; (**b**) cover thickness and (**c**) concrete strength.

A cross-section in the mid-span of a RC beam is taken as an example to elaborate work phases during the whole test, as shown in Figure 8. Figure 9 presents the relationship between crack width on top-surface and crack depth on side-surface of beams. The work phase of a cross-section of concrete can be classified into three phases.

Phase Ⅰ: Uncracked phase.

Initially, the moment (*M*) applied on the RC beam was far less than cracking moment (*M_cr_*), and the stress of concrete (*σ_c_*) varied linearly with the height of the beam (Figure 8b). The deformation of structures could be attributed to the elastic deformation of aggregates and cement crystals. When the moment approaching to cracking moment, the stress of concrete near the surface of tensile zone presented plastic characteristic. The neutral axis barely moved in this stage.

Phase Ⅱ: Crack propagation phase.

The tensile stress was always being undertaken by both reinforcements (*σ_s_*) and concrete (*σ_c_*) together before cracking. Once the first crack emerged on the top surface, the balance of forces would be broken, which resulted in uneven strain existing in reinforcements and concrete, inducing debonding of the reinforcements. The loss of local bonding stress would decrease the members’ stiffness and accelerate cracking. That is the reason why a tiny increase in crack width could lead a great extension in depth crack, as shown in Figure 9. The first cracking loads of specimens were listed in Table 6. In this stage, the moment was not sufficiently large to make reinforcement yielding (*M_y_*). The stress of concrete was completely in plastic-statement and the neutral axis moved rapidly to the compression zone (Figure 8d). The crack depth approximately ranged from 0 to 150 mm (Figure 9).

Phase Ⅲ: Fracture phase.

When longitudinal reinforcements reached the yielding strength (*f_y_*), the tensile stress of concrete (*σ_c_*) tailed away (Figure 8e,f). As the moment continued increasing to the ultimate moment (*M_u_*) (*M_y_/M_u_* = 75–85% in this paper), the crack depth ranged from 150 to 250 mm. The bond between the reinforcement and concrete was almost lost due to cracking, and nonlinear behavior was observed between the crack width and crack depth (Figure 9). Finally, the RC beam failed with concrete crushing in the compression zone. Crack depth didn’t experience obvious changes as a result of the limitation from the compression zone and the beam height.

**Figure 10:**
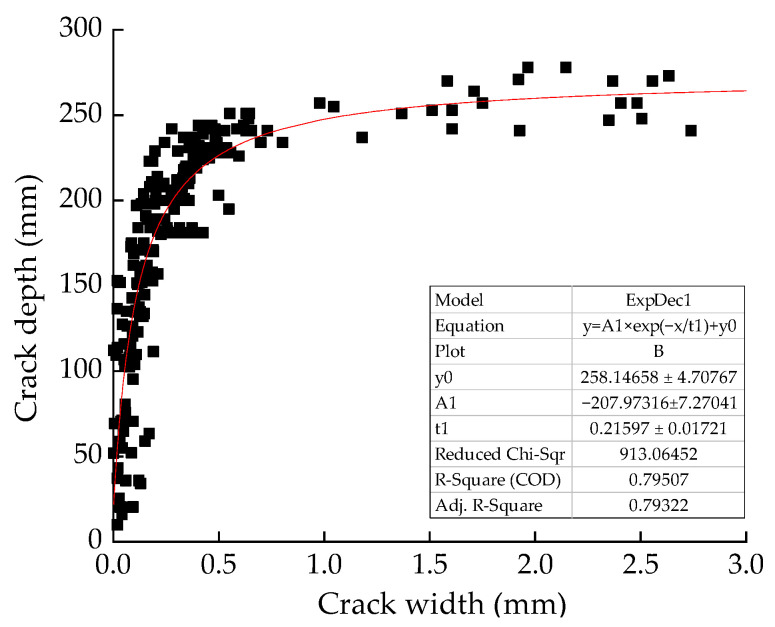
Curve fitting on crack depth and width.

Equation (5) reveals the relation between crack width (*w*) and crack depth (*h_c_*). We found the analysis would be carried out well by fitting experimental data with exponential function, as shown in Figure 10. The fitting equation only works for the RC beam with a height of 300 mm and was subject to a bending moment.

(5) $$h_{c}=258-\frac{208}{1+\exp(w/0.216)}.$$

The position of the neutral axis influences the development of the crack depth, while the position and moving rate of the neutral axis are governed by the beam height and also by the bending moment. As analyzed above, it can be reasonably assumed that higher cross-section of beams means higher tension zone, which makes crack depth develop at a relative faster rate. Limited by experimental conditions, the RC beams only with 200 × 300 mm^2^ cross section were analyzed in this paper. The section height wasn’t taken into account as a variable for studying the correlation.

### 4.2. Verification of the Fitting Function

As the limitation abovementioned in the previous section, Equation (5) is applicable for RC beams (with a height of 300 mm) only subjected to a bending moment. Rimkus [29] and Gribniak [30] conducted bending tests of nine beams reinforced with glass fibre reinforced polymer (GFRP) or steel bars. They marked the value of loads alongside crack depth extension during tests procedures. Beam S1-1, S1-2 and S1-4 were randomly selected to illustrate the predictive of Equation (5) (Figures 11, 12, 15 in literature [29]). Cracks with the maximum width were taken as examples. Since the authors did not directly present the information we need, such as crack width on the surface, key steps are necessary to be followed to obtain the basic values of parameters. For uniformity between model in proposing and verification, the criterion involved in calculation are still the chosen formulas advised in GB [18] (refer to Table 1, Equations (2) and (3)).

Key steps of verification include: (1) based on the loading system and characteristic parameters of beams mentioned in literature [29,30] (as shown in Table 9), the stress of reinforcement (*σ_sq_*) can be figured out; (2) the maximum crack width (*w*^0^_max_) on the surface of concrete can be obtained on the basis of GB [18]; (3) the theoretical crack depth (*h^0^_c_*) can be forecasted by Equation (5); (4) comparing theoretical and actual figures of crack depth.

Table 10 presents theoretical (*h*^0^*_c_*) and actual (*h_c_*) values of crack depth in S1-1, S1-2 and S1-4 under different loads. The variations are 2.6, 3.4, and 2.3%, respectively. The conclusion can be drawn that the fitting function proposed in this paper is in good agreement with previous work.

## 5. Summary and Conclusions

Cracks propagating inside RC components are barely able to be detected by visual inspection. This paper aims to find a proper way to assess cracking depth without any help from equipment. The correlation between crack width on the surface and crack depth was investigated by carrying out a bending test on eight RC beams. The influence of variables regarding concrete type, cover, and reinforcement ratios were taken into consideration. The main conclusions of the present study are as follows:

1. The cracking load of specimens is 30 ± 4 kN, and the splitting tensile strength of concrete has a certain influence on the cracking load. In the early stage, the maximum crack width increased slowly with the applied load. When the load was up to 75–85% of the ultimate loading capacity, the turning point arose and then the growth rate of crack width became faster. The relationship between crack width and load was generally linear for all specimens in the whole loading process, even after yielding of the longitudinal reinforcements.
2. The crack initiation on side-surface was later than its appearance on top-surface of beams. But the crack depth propagated more quickly, reaching the original position of neutral axis in a very short time. After that, the rate became slower due to the limitation from the compression zone.
3. The theoretical analysis of correlation between crack width and crack depth was carried out by combining work phases of a cross-section of concrete and curves of depth/width. The work phase can be classified into three phases, namely uncracked, crack propagation, and fracture phases. The curves of depth/width grew linearly in the beginning. After that, no linear behavior was observed. When the reinforcements yielded, the crack depth remained stable even with a great increase of crack width.
4. On a foundation of achievements in this paper, an exponential function was proposed to predict load-induced crack depth. Verification work has been conducted to illustrate that the fitting function achieved a good agreement between the theoretical and the experimental relationship.

## Figures and Tables

**Table 1:** Calculations for crack width.

Standard	Calculation of Crack Widths	Parameter
EN (Comprehensive theory)	$w=S_{r,\max}(\varepsilon_{sm}-\varepsilon_{cm})$, $\varepsilon_{sm}-\varepsilon_{cm}=\frac{\sigma_{s}-k_{t}\frac{f_{ct,eff}}{\rho_{p,eff}}(1+\alpha_{e}\rho_{p,eff})}{E_{s}}$.	*w* is crack width, *S_r,_*_max_ is the maximum crack spacing; *ε_sm_* is the mean strain in the reinforcement; *ε_cm_* is the mean strain in the concrete between cracks; *σ_s_* is the stress in the tension reinforcement; *k_t_* is a factor dependent on the duration of the load; *α_e_* is average tensile strength of concrete when cracks are about to appear; *ρ_p,eff_* is effective reinforcement ratio. *E_s_* is elastic modulus of reinforcement.
ACI (No-slip theory)	$s\leq 15(\frac{40,000}{f_{s}})-2.5c_{c}$, $s\leq 12(\frac{40,000}{f_{s}})$. $(w_{c}=2\frac{f_{s}}{E_{s}}\sqrt{d_{c}^{2}+d_{s}^{2}}$. [17])	*s* is the spacing between reinforcement; *f_s_* is the stresses in reinforcement; *c_c_* is the clear cover of reinforcement. (*w_c_* is the maximum crack width; *E_s_* is elastic modulus of reinforcement; *d_c_* is the distance from the concrete bottom to the centroid of the reinforcement near the bottom; *d_s_* is the shortest distance from the side edge of concrete to the centroid of the edge reinforcement)
GB (Comprehensive theory)	$w_{\max}=\alpha_{cr}\psi \frac{\sigma_{sq}}{E_{s}}(1.9c_{s}+0.08\frac{d_{eq}}{\rho_{te}})$.	*w*_max_ is the maximum crack width; *α_cr_* is characteristic coefficient of members; *ψ* is the uneven strain coefficient of tensile steels; *σ_sq_* is the tensile stress in longitudinal reinforcement; calculated under the quasi-permanent combination of actions; *c_s_* is the distance from the outer edge of the outermost tensile steel bar to the edge of the tension zone, *d_eq_* is the equivalent diameter of the longitudinal rebar; *ρ_te_* is viewed as effective reinforcement ratio.

**Table 2:** Exposure classes related to environment conditions and the maximum allowable crack width.

Description of the Environment	No Risk of Corrosion or Attack	Corrosion Induced by Carbonation	Corrosion Induced by Chlorides	Corrosion Induced by Chlorides from Marine	Freeze/Thaw Attack	Chemical Attack
EN *w_cr_* (mm)	X0 0.4	XC1–XC2 0.4	XD1–XD3 0.3	XS1–XS3 0.3	XF1–XF4 0.3	XA1–ZA3 0.3
GB *w_cr_* (mm)	Ⅰ 0.3	Ⅱa–Ⅱb 0.2	Ⅱb 0.2	Ⅲa 0.2	Ⅲa–Ⅲb 0.2	Ⅲb 0.2

**Table 3:** Mix proportions of concrete (kg/m^3^).

Concrete Type	Water	Cement	Sand	Coarse Aggregate	Water/Cement
C20	170	300	732	1193	0.57
C30	180	334	660	1228	0.54
C40	180	408	562	1250	0.44

**Table 4:** Material properties of concrete and rebar (MPa).

Concrete Type	Compressive Strength (*f_cu_*)	Tensile Strength (*f_tk_*)	Rebar Diameter (mm)	Yield Strength (*f_y_*)	Ulitimate Strength (*f_su_*)
C20	38.1	2.70	Ø 22	446	686
C30	44.1	2.98	Ø 18	564	691
C40	48.0	3.07	Ø 14	544	728

**Table 5:** Properties of the beams (mm).

Specimen ID	Concrete Type	Cover Thickness	Longitudinal Reinforcement	
			N & d ^2^	*ρ_eff_*
A1, A2 ^1^	C30	35	2 Ø14	0.616%
B	C30	35	2 Ø 22	1.52%
C	C30	35	2 Ø 18	1.03%
D	C30	25	2 Ø 14	0.616%
E	C30	45	2 Ø 14	0.616%
F	C20	35	2 Ø 14	0.616%
G	C40	35	2 Ø 14	0.616%

^1^ A was reference beam, we made two of it and marked with A1 and A2, separately. ^2^ N & d denote rebar number and rebar diameter, respectively; *ρ_eff_* is effective reinforcement ratio.

**Table 6:** Cracking loads of beams (kN).

Specimen ID	A1	A2	B	C	D	E	F	G
The first cracking load	30	30	26	30	32	34	30	34
The second cracking load	46	44	44	44	46	46	48	48

**Table 7:** Variables involved in computation.

Parameter	*w_s_*_,max_ (mm)	*τ_l_*	*α_cr_*	*E_s_* (MPa)	*c_s_* (mm)	*d_eq_* (mm)	*b* (mm)	*h* (mm)
**Value**	0.3	1.5	1.9	2 × 10^5^	33, 43, 53	14, 18	200	300

**Table 8:** Comparison between different methods.

Specimen ID	A1	A2	C	D	E	F	G
*ε* (×10^−6^)	1700.9	1691.2	1617.5	1851.4	1600.1	1591.2	1796.9
*σ_a_* (MPa)	340.2	338.2	323.5	370.3	320.0	318.2	359.4
*σ_sq_* (MPa)	341.4	341.4	328.5	366.4	321.0	314.3	363.3

**Table 9:** Variables involved in computation.

Beam	Load (kN)	*α* *_cr_*	*f_tk_* (MPa)	*A_s_* (mm)	*ρ_te_(*%)	*c* (mm)	*d_eq_* (mm)	*h_c_* (mm)
S1-1	128	1.9	3.0	695.9	1	20	10	213.5
S1-2	110			776.8			14	236.6
S1-4	52			760.0			22	166.0

**Table 10:** Comparation between theoretical and actual values of crack depth.

Beam	Load (kN)	*w*^0^_max_ (mm)	*h*^0^*_c_* (mm)	*h_c_* (mm)	Variation (%)
S1-1	128	0.281	213.5	219.1	2.6
S1-2	110	0.468	236.6	228.8	3.4
S1-4	52	0.050	166.0	162.2	2.3

## Data Availability

Data sharing not applicable.
